# Polyproline type II helical antifreeze proteins are widespread in Collembola and likely originated over 400 million years ago in the Ordovician Period

**DOI:** 10.1038/s41598-023-35983-y

**Published:** 2023-06-01

**Authors:** Connor L. Scholl, Martin Holmstrup, Laurie A. Graham, Peter L. Davies

**Affiliations:** 1 Department of Biomedical and Molecular Sciences, Queen’s University, 18 Stuart Street, Kingston, ON K7L3N6 Canada; 2 Section of Terrestrial Ecology, Department of Ecoscience, Aarhus University, C.F. Møllers Allé 4, 8000 Aarhus C, Denmark; 3 Arctic Research Center, Aarhus University, Ny Munkegade 114, 8000 Aarhus C, Denmark

**Keywords:** Molecular evolution, Proteins

## Abstract

Antifreeze proteins (AFPs) bind to ice crystals to prevent organisms from freezing. A diversity of AFP folds has been found in fish and insects, including alpha helices, globular proteins, and several different beta solenoids. But the variety of AFPs in flightless arthropods, like Collembola, has not yet been adequately assessed. Here, antifreeze activity was shown to be present in 18 of the 22 species of Collembola from cold or temperate zones. Several methods were used to characterize these AFPs, including isolation by ice affinity purification, MALDI mass spectrometry, amino acid composition analysis, tandem mass spectrometry sequencing, transcriptome sequencing, and bioinformatic investigations of sequence databases. All of these AFPs had a high glycine content and were predicted to have the same polyproline type II helical bundle fold, a fold unique to Collembola. These Hexapods arose in the Ordovician Period with the two orders known to produce AFPs diverging around 400 million years ago during the Andean-Saharan Ice Age. Therefore, it is likely that the AFP arose then and persisted in many lineages through the following two ice ages and intervening warm periods, unlike the AFPs of fish which arose independently during the Cenozoic Ice Age beginning ~ 30 million years ago.

## Introduction

Organisms living in sub-zero environments must adapt to avoid cellular injury due to freezing. Formation of ice crystals within living tissues poses the risks of cell dehydration and the rupture of cellular membranes, leading to death^1^. Organisms occupying niches that experience sub-zero temperatures often produce antifreeze proteins (AFPs), which function by adhering to, and preventing the growth of, ice crystals^2^. Once bound, ice growth is limited to areas around the AFP, causing micro-curvatures to form on the ice surface^3,4^. This makes it energetically unfavourable for water to join the ice lattice resulting in a depression of the freezing temperature below the melting temperature, which is termed thermal hysteresis (TH) and is used to quantify an AFP’s potency.

The first AFP characterized was from a teleost fish^5^. Since then, AFPs have been observed in other fishes^6^, insects^7,8^, and microorganisms^9–11^. In fish, AFPs are thought to have arisen during the Cenozoic era beginning 20–40 million years ago when sea ice at the poles was present for the first time in ~ 200 million years^12,13^. In seawater, the high concentration of NaCl (~ 0.45 M) depresses the freezing temperature of seawater to ~ − 1.9 °C. As fish blood has a lower solute concentration and freezes at ~ − 0.8 °C, any contact with ice crystals could nucleate freezing and kill the fish^5^. Consequently, fish AFPs must be produced in adequate quantities and with sufficient activity to decrease the body freezing temperature by at least 1.1 °C. This provides a selective advantage to these fish, as they can safely hunt for food in ice-laden seas where fish lacking AFPs are at risk of freezing. There have been four types of AFPs found in fish: (1) alanine-rich type I AFP, (2) lectin-like type II AFP, (3) type III AFP derived from sialic acid synthase, and (4) antifreeze glycoproteins (AFGPs). With different folds that all perform the same function, it raises the question of how these AFPs first arose^14^.

Alanine-rich alpha-helical type I AFPs have independently evolved on at least four occasions^15^. The simple repetitive AFGPs have arisen on two independent occasions including once from a trypsinogen gene by duplication and divergence^16^. Type II AFPs have evolved from a C-type lectin progenitor and have been spread to at least two distant taxonomic branches of fish by lateral gene transfer^17,18^. Duplication and divergence of a sialic acid synthase gene has given rise to the type III AFP gene family, which has only been found in one branch of fishes^19^. It is thought that these fish AFP folds have arisen within the last few tens of millions of years in response to polar glaciation^20^. In other branches of organisms like insects^21,22^ and microorganisms^23,24^ there are also examples where different AFP folds have independently arisen to perform the same task.

Collembola are the most abundant terrestrial arthropods and are found on all continents^25^. These small organisms (most are commonly only a few millimeters long) are typically soil dwelling, but some species live in trees, on ponds or wet surfaces surrounding stones, or on glaciers. Collembola got their name from an abdominal organ, the collophore, which is involved in osmo- and ion regulation^26^. A second defining abdominal anatomical feature is a spring-loaded organ known as a furca, which allows them to escape predators by “jumping”. This has given them their nickname “springtails”. These primitive organisms arose around 450 million years ago and there are close to 10,000 species classified to date^27^. Those that live in sub-zero environments have cold tolerance mechanisms to survive in these harsh conditions, one of which is the production of AFPs. Previously a few species of Collembola have been found to have TH activity^28–31^, and cold hardiness and supercooling abilities^32^ but no systematic study of the AFP types present and their distribution in Collembola has been made to date.

The first collembolan AFP characterized was the small 6.5-kDa isoform from *Hypogastrura harveyi* (*Hh*AFP)^29^. *Hh*AFP was predicted to have a glycine-rich polyproline type II (PPII) helical bundle fold^33^ a structure that was later confirmed by X-ray crystallography^34,35^. This fold, which appears unique to Collembola, consists of two layers of antiparallel PPII helices connected by loop regions. Each rotation around the helix is exactly three residues in length with tripeptide repeats of G-X_1_-X_2_, where X_1_ is often glycine. The core of the protein contains the inward facing glycine residues, allowing for tight packing due to the absence of side chains^33^. Compact packing of the helices allows for a hydrogen bonding network to develop between the helix backbones that increases the stability of the fold^36^. The two layers both have an outward pointed face. One surface is flat, contains small, hydrophobic residues, and is thought to function as the ice-binding surface (IBS)^37^. This surface is alanine-rich, but also contains serine, threonine, and valine residues. The opposing surface is uneven and contains larger residues, some of which are polar or charged. *H. harveyi* also produces a larger 15.6-kDa AFP isoform that is proposed to have 13 polyproline helices^38^.

A putative homolog of *Hh*AFP was recently characterized from *Megaphorura arctica* (*Ma*AFP) collected in Iceland^31^. Although the 6.5-kDa *Ma*AFP shares high similarities in coding sequence to *Hh*AFP, their untranslated regions (UTRs) are divergent to the extent that common ancestry could not be definitively established. Additionally, 9.6-kDa AFP isoforms from *Granisotoma rainieri* (*Gr*AFP) were studied^39^. The structure of *Gr*AFP was modelled to be a polyproline type II bundle with nine helices, which was confirmed using X-ray crystallography. When the UTRs of the five *Gr*AFP cDNAs were compared, the most dissimilar shared only 69% sequence identity. Therefore, a lack of similarity between the UTRs of different species does not disprove homology.

Here we have characterized the AFPs from 18 species of Collembola from numerous families within two of the four extant orders of Collembola, collected from four different continents. The extent of the analysis was determined by the amount of biomass available. Small quantities of springtails (< 100 mg of freeze-dried tissue) were used to determine TH activity and ice-shaping. AFPs were purified from larger samples (> 100 mg of freeze-dried tissue) by ice affinity purification (IAP) and characterized by MALDI-MS, amino acid composition, and/or tandem mass spectrometry. Transcriptomes were generated from some species to deduce AFP sequences at the nucleic acid level, and in some cases to recombinantly express the encoded proteins. AFPs present in the different Collembola tested here all inhibited ice growth on the basal plane, suggesting that they could be hyperactive. Where more detailed analysis was possible, all the AFPs examined had the same glycine-rich tripeptide repeating pattern indicative of the PPII helical bundle. To date, this fold has only been found in Collembola and its presence across distant species suggests that the PPII helical bundle fold originated in a basal collembolan species, shortly after the group arose.

## Materials and methods

### Collembola collection

Here, 20 new species representing five families were tested for TH (Supplementary Table 1). Most species were collected in the field by the authors, whereas a few were supplied from cultures of other laboratories. Collembola were typically maintained for several years in Petri dishes with moist plaster of Paris mixed with charcoal, and fed dried baker’s yeast and/or green algae ad libitum. All species were kept at 20 °C with 12 h light and 12 h darkness. Exceptions from this procedure were *M. arctica* and *Entomobrya nivalis*, that were field collected and cold acclimated in the laboratory (Supplementary Table 1), and *Cryptopygus antarcticus* that were frozen immediately after being field collected. *H. harveyi*^29^ and *G. rainieri*^39^*,* which completed the study group of 22 species, were collected as previously described.

### Sample preparation

To induce AFP synthesis, specimens were acclimated in darkness and cold at 10 °C for 19 days, followed by 5 °C for 13 days, and 1.5 °C for 28 days. Animals were then freeze-dried for 2 days. Dried animals were added 1:8 (w/v ratio) to buffer (50 mM Tris–HCl (pH 7.8), 150 mM NaCl, 1 mM phenylthiocarbamide and 1 × EDTA-free Roche protease inhibitor cocktail) and homogenized by hand using a disposable plastic pestle in a 1.5-mL microcentrifuge tube. All manipulations and temporary storage of samples were done on ice or at 4 °C to prevent thermal denaturation of the AFP. The homogenates were centrifuged at 16,300×*g* for 30 min at 4 °C. The aqueous fraction beneath the lipid layer was removed for TH measurements.

### Antifreeze protein isolation

AFP extractions for protein characterization were performed using > 100 mg of freeze-dried animals. Tissue samples were homogenized in buffer (50 mM Tris–HCl (pH 7.8), 150 mM NaCl, 1 mM phenylthiocarbamide and 1 × EDTA-free Roche protease inhibitor cocktail) using an IKA ULTRA-TURRAX disperser (Staufen, Germany). The homogenate was centrifuged at 22,000×*g* for 30 min and the supernatant was filtered through glass wool to remove lipid. AFPs in the filtered supernatant were recovered using four rounds of ice-shell purification as previously described^40^. The final ice fraction for each preparation was concentrated to < 500 µL using an AmiconUltracel 3 K filter (MilliporeSigma, Burlington, MA, USA) spun in a Sorvall ST16R centrifuge at 3000×*g*.

### Amino acid analysis

Amino acid compositions were determined at the SickKids Proteomics, Analytics, Robotics & Chemical Biology Centre (SPARC, The Hospital for Sick Children, Toronto, ON, Canada) by acid hydrolysis as previously described^40^ and were also used to calculate protein concentration.

### Mass spectrometry

MALDI-TOF MS was performed at the Protein Function Discovery Facility (Queen’s University, Kingston, ON, Canada) using alpha-cyano-4-hydroxycinnamic acid matrix on dried droplets with a SCIEX Voyager DE Pro in Linear mode. Protein sequencing by tandem mass spectrometry was done at the SickKids Proteomics, Analytics, Robotics & Chemical Biology Centre (SPARC, The Hospital for Sick Children, Toronto, ON, Canada).

### RNA extraction and transcriptome sequencing

RNA samples were extracted from 30 to 50 mg of tissue stored in RNAlater (Thermo Fisher, Waltham, MA, USA). Extraction was performed as previously described^39^. The transcriptome of *C. antarcticus* was assembled at the Sequencing & Genotyping Center (University of Delaware, Newark, DE, USA), and those of *G. rainieri* and *E. nivalis* were assembled at the Institute for Genome Sciences (University of Maryland, Baltimore, MD, USA).

### Thermal hysteresis measurements

The AFP-containing samples were injected into a grid filled with immersion oil on a Peltier unit. The temperature was controlled using a nanoliter osmometer (Micro-Ice Ltd, Alon Shvut, Israel) and a model 3040 temperature controller (Newport, Irvine, CA, USA). The samples were flash-frozen and melted slowly until a single ice crystal remained. The temperature was held just below the melting point and then decreased at a rate of 0.075 °C/min until ice growth began. Videos of the ice crystals during TH measurements were recorded either using Panasonic WV-BL200 CCTV camera or a DMK 33UX249 USB 3.0 monochrome industrial camera (The Imaging Source, Charlotte, NC, USA).

### Gene synthesis and expression

Codon-optimized, synthetic genes for *G. rainieri* (QQY00623.1) and *Folsomia candida* (OXA44825.1) AFPs were ordered from GeneArt (Thermo Fisher, Waltham, MA, USA). The DNA encoding the signal peptide was removed and an N-terminal methionine residue was encoded to help introduce an NdeI cut site. To the C terminus, leucine and glutamate codons were introduced to add a XhoI cut site. The genes were subcloned into pET-24a vectors^39^. The resulting plasmids were transformed into TOP10 competent cells (Invitrogen, Carlsbad, CA, USA) for isolation using a GeneJET Plasmid Miniprep kit (Thermo Fisher, Waltham, MA, USA). DNAs were sequence checked before the plasmids were retransformed into BL21 (DE3) expression cells (Invitrogen, Carlsbad, CA, USA). Cell cultures were grown in lysogeny broth medium with 100 µg/mL kanamycin at 37 °C. Upon reaching an OD_600_ of 0.6–0.8 the cell cultures were cooled to 20 °C, and 1 mM isopropyl β-d-1-thiogalactopyranoside was added to induce the cell culture overnight. Cells were centrifuged at 4500×*g* for 30 min and resuspended in 50 mL of lysis buffer (20 mM Tris/HCl (pH 7.8), 500 mM NaCl, 5 mM imidazole, 0.1 mM phenylmethylsulfonyl fluoride, and one dissolved tablet of cOmplete™ ultra protease inhibitor cocktail). Cells were sonicated 16 times at 10 s per round and cooled to 4 °C between cycles to prevent protein denaturation.

### Recombinant protein isolation

His-tagged recombinant AFPs were separated from the lysate supernatant using Ni-affinity chromatography. Fractions containing AFP were pooled, loaded into a 250-mL round-bottom flask seeded with an ice shell, and ice-affinity purified^39^.

### Phylogenetic tree assembly

The taxonomy IDs for each species were extracted from the NCBI taxonomy database. The phylogenetic tree was assembled using phyloT (https://phylot.biobyte.de/).

## Results and discussion

### Antifreeze activity is present in multiple genera of Collembola

**Figure 1:**
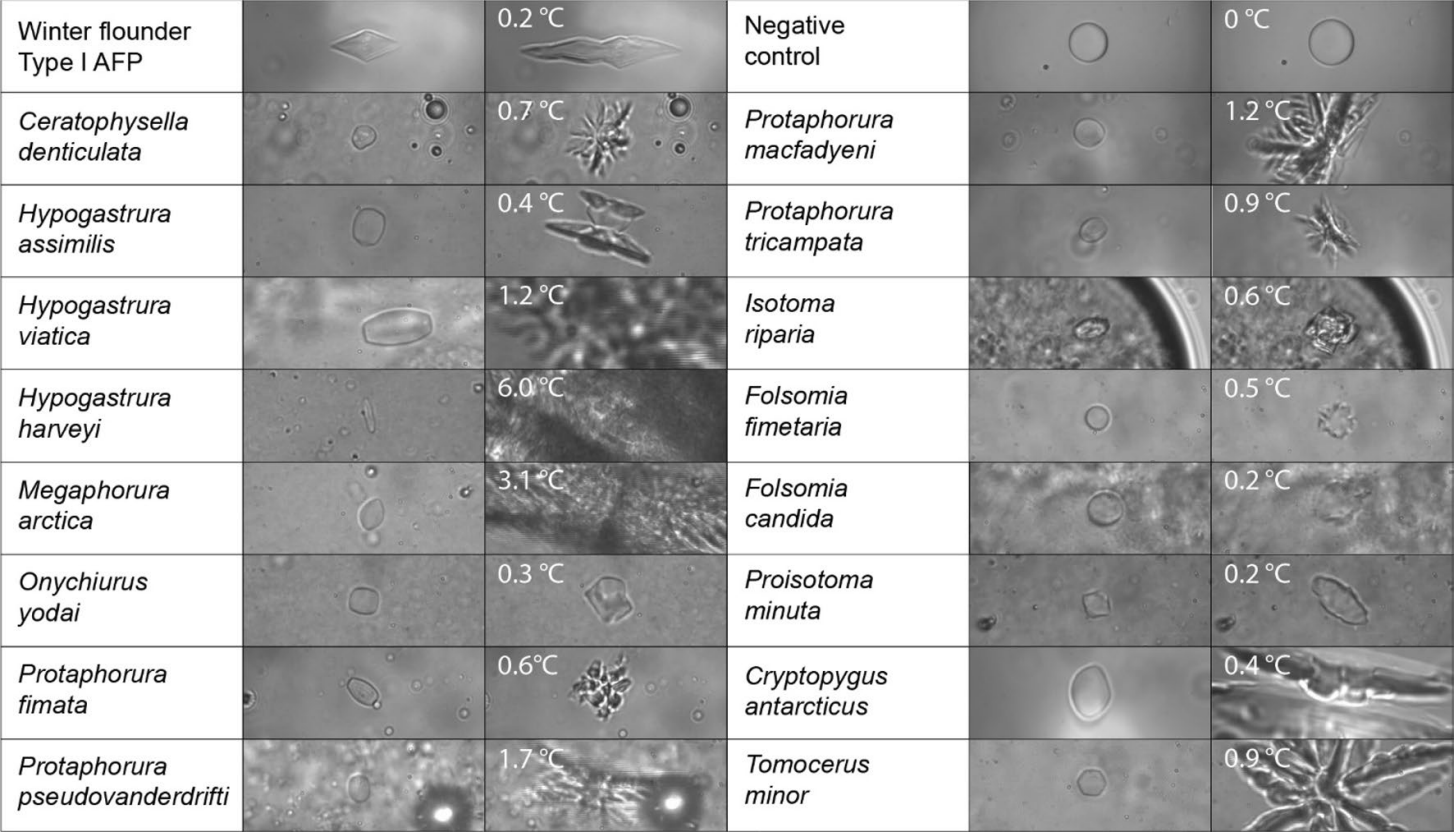
Comparison of ice-shaping in collembolan homogenates. Freeze-dried Collembola were gently homogenized in buffer (8:1 v/w) and the supernatants were assayed for antifreeze activity (TH). For each species (left column), an image was captured from the video during the TH measurement (middle column) and just as the freezing point was exceeded (right column). The positive control is type I AFP from winter flounder (*Pseudopleuronectes americanus*). Negative control is buffer (50 mM Tris–HCl (pH 7.8), 150 mM NaCl, 1 mM phenylthiocarbamide and 1 × EDTA-free Roche protease inhibitor cocktail).

Whole homogenate supernatants from 22 different species of Collembola were assessed for TH activity and ice crystal shaping (Fig. 1). Of the 22 species tested, 18 had TH activity. The single ice crystals monitored in active homogenates melted into defined oblong shapes that were symmetrical around the *c*-axis, suggesting that the AFPs are binding to and stabilizing several ice planes, including the basal plane. In contrast, crystals formed in the presence of an AFP that does not bind to the basal plane, namely type I AFP, melt into discs that grow into hexagonal bipyramids as they are being cooled (Fig. 1, top left panels). When the freezing point was exceeded in collembolan samples, dendritic ice growth emanated from the *a*-axes and grew rapidly in samples that had high TH activity. In *Hypogastrura viatica* and *M. arctica* the dendritic burst covered the field-of-view within one frame (1/12 s). In contrast, ice crystals in the buffer control were disk-shaped and kept growing with the same shape, while the burst with type I AFP occurred along the *c*-axis (Fig. 1 top right panels). Samples were homogenized at the same w/v ratios making comparisons of relative TH activity possible, and the activity in different species ranged from 0.2 to 1.7 °C. These differences could arise from variation in gene copy number, expression levels and/or the activity of the AFPs.

Fluorescent ice plane analysis of the large *H. harveyi* AFP isoform showed binding to both basal and prism planes of ice^38^. Support for basal plane binding was also provided by the X-ray crystal structure of *Gr*AFP, as the crystallographic waters could be aligned to both the basal and primary prism planes of ice^39^. In addition, the high activity (> 2 °C) of two of the homogenates suggest that, as in other arthropods such as *Tenebrio molitor*^41^, most Collembola produce hyperactive AFPs. Additionally, the consistent differences in ice shaping and the burst between collembolan AFPs, and type I AFP from winter flounder (Fig. 1) is likely due to basal-plane binding by the collembolan AFPs.

### Multiple springtail species produce glycine-rich AFPs

**Figure 2:**
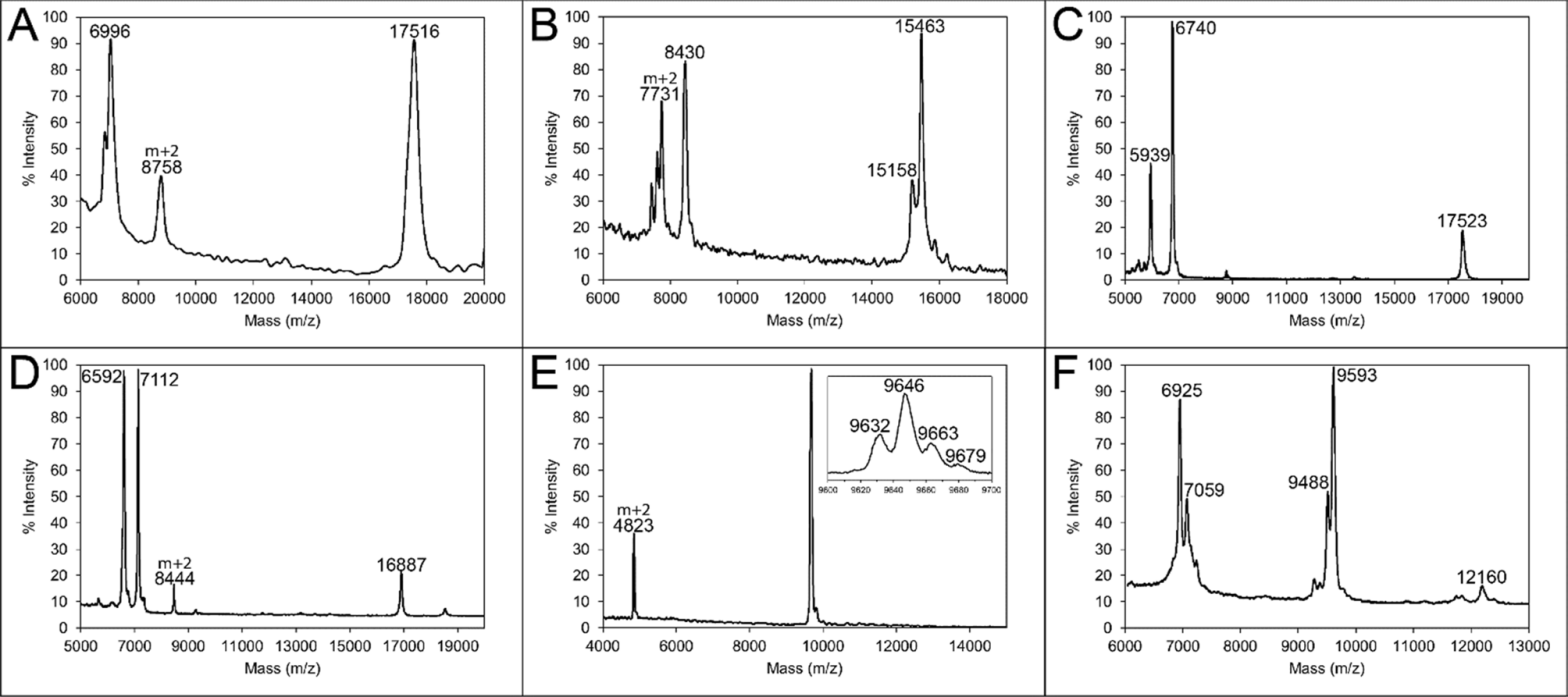
MALDI spectra of purified collembolan AFP extracts. Proteins from: (**A**) *Protaphorura pseudovanderdrifti*, (**B**) *Cryptopygus antarcticus*, (**C**) *Ceratophysella denticulata*, (**D**) *Megaphorura arctica*, (**E**) *Folsomia candida*, and (**F**) *Granisotoma rainieri* were purified with four rounds of ice-affinity purification and subjected to MALDI-MS. Major peak masses are labelled.

Amino acid compositions of AFPs extracted from three species of Collembola (*H. harveyi*, *G. rainieri*, and *M. arctica*) have been previously reported^29,31,39^. Here, AFP extracts from three additional species (*Cryptopygus antarcticus*, *Folsomia candida*, and *Protaphorura pseudovanderdrifti*) were also subjected to amino acid analysis (Supplementary Table 2). All had high abundances of glycine and alanine, which are diagnostic of the PPII helical bundle fold. However, these glycine and alanine proportions were lower than for *H. harveyi*, which had been further purified, suggesting some contamination by trace levels of other proteins. This is to be expected as each round of IAP only reduces non-AFP protein levels ~ 10 fold^40^. Nevertheless, MALDI-MS suggested that the AFPs were the dominant species after IAP (Fig. 2). *P. pseudovanderdrifti* (Fig. 2A), *C. antarcticus* (Fig. 2B), and *Ceratophysella denticulata* (Fig. 2C) extracts all showed a few discrete peaks, corresponding to two or more small isoforms (5.9–8.8 kDa) and one or more large isoforms (15.5–17.5 kDa), similar to what was previously reported for *M. arctica* (Fig. 2D)^31^ and *H. harveyi* (6.5 and 15.7 kDa)^29^. In contrast, *F. candida* has one main peak consisting of four sub-peaks that differ by ~ 16 Da (Fig. 2E) and *G. rainieri* (Fig. 2F)^39^ has three clusters of isoforms within a narrower range of 6.9–12.2 kDa. There also appear to be small variations in the isoforms as indicated by the shoulder peaks. Much like the five 9.6-kDa isoforms from *G. rainieri*^39^, there are likely isoforms within each population with a few amino acid polymorphisms.

### AFP sequence determination

Partial sequencing of the purified AFPs by tandem mass spectrometry of tryptic fragments provides a robust link between the isolated proteins and their nucleic acid sequences. This has previously helped deduce full-length *M. arctica* and *G. rainieri* AFP sequences from transcriptome data^31,39^. In this study, tryptic fragments of *C. antarcticus* and *F. candida* AFPs were also rich in glycine and alanine and contained GXX repeat motifs (Table 1).

**Table 1:** Tryptic fragment sequences of *Cryptopygus antarcticus* and *Folsomia candida* AFPs determined by tandem mass spectrometry.

Mass (Da)^a^	Sequence^b^	Isoform match
1953.8	(CA)NGQSGGAGGTGGAGSAGGTCAGK	*Ca*AFPa-1
1921.8	(CT)DGANGANGADGAAGGGFPGGK	*Ca*AFPa-2,5,6,7
1118.5	(GA)DGAAGGGFPGGK	*Ca*AFPa-1–7
1960.9	(AGA)ANTNGNGGIGGAGGAGGACGTK	*Ca*AFPb-1–4
699.4	(GG)VGAPGGK	*Ca*AFPb-5–7
747.3	(pQC)TTGVR^c^	*Ca*AFPb-5–7
7206.2	GANGVSGITGAAGIGGPGGQPGAGGQGGACYGAFNGGVGGHGGNGGAAGAGQSGPNGADGGAGGDA | GPSG | N | GG | AGGYGGHGGASGASGASK^d^	*Ca*AFPb-5–7
1210.6	(GA)DGPVGASSGAHK	*Ca*AFPb-8
1336.6	(GA)GGYGGAGGAGGAAGTK^e^	*Ca*AFPb-8
1504.7	(GA)GGYGGAGGAGGAAGTKPA	*Ca*AFPb-8
1515.7	AGTAGTHGGFGGAGGAGGVG	*Ca*AFPb-8
1618.8	(SN)GIVGNAGGTGCNGGVGK	*Ca*AFPb-8
1945.9	(PA)GIGAAGGNGGNAGTGGAGGYGGK^e^	*Ca*AFPb-8
2303.0	(GGA)GGASGTCNGHDGAAGGAGGVGDSGTK	*Ca*AFPb-8
2494.1	(CFGTHNGGAGGA)GAPGGAASSAHDGPAGGK^f^	*Ca*AFPb-8
3263.5	(GAG)GYGGAGGAGGAAGTKPAGIGAAGGNGGNAGTGGAGGYGGK	*Ca*AFPb-8
2493.0	(CS)GAAGADGTSNGQAGLAGTAGG**P**GCDGGR^f^	*Fc*AFP
3192.4	(SGTGTG)GHGGNGGDGH**P**GGA(**P**G)AGGA(PN)GL (**P**G)SA(GNTLP)^f^	*Fc*AFP
3979.8	(GGAG)F**P**GTNVPGGAGGAGGAGGSGNTAGGAGGHGGSSNTLTGGAGGAGGIR^f^	*Fc*AFP

^a^Monoisotopic [M+H]+. ^b^Residues in brackets were consistent with the mass of the lightest b-ion observed. ^c^Fragment mass consistent with N-terminal Gln cyclization (pyroglutamate). ^d^Fragment masses exceeded instrument cutoff, but in-source fragmentation generated a series of C-terminal fragments beginning at Lys and extending to the residues delineated with |. ^e^Fragments resulting from cleavage of 3263.5 Da product between Lys and Pro. ^f^Bolded proline residues were seen to be occasionally hydroxylated.

**Figure 3:**
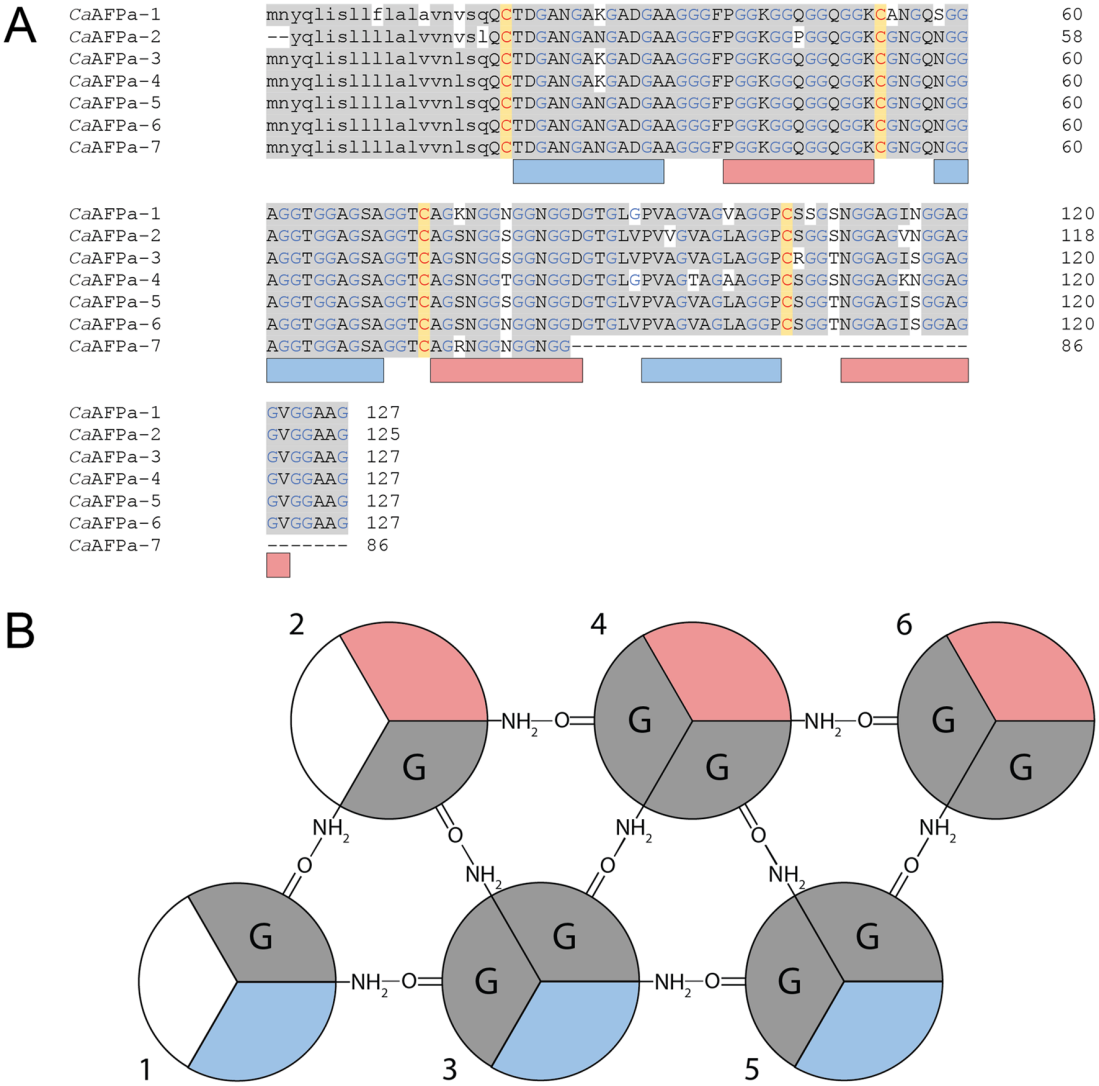
Sequence alignment of the 8.5-kDa *Ca*AFP isoforms. (**A**) The amino acid sequences of *Ca*AFP isoforms from ESTs and the generated transcriptome were aligned. Identical amino acid residues are highlighted in grey. Glycine residues are coloured blue. Cysteine residues are coloured red and highlighted yellow. The signal peptides are shown in lowercase and the coding sequence is shown is uppercase. The rectangles below show putative PPII helices for the IBS (blue) and non-IBS (red) faces. *Ca*AFPa-1, OQ445586; *Ca*AFPa-2, GR870234; *Ca*AFPa-3, OQ445587; *Ca*AFPa-4, FF279148; *Ca*AFPa-5, OQ445583; *Ca*AFPa-6, GR869204; *Ca*AFPa-7, FF278983. (**B**) Schematic of six polyproline type II helical bundle. The antiparallel helices are connected by loop regions (not shown) and arrange into two layers. The ice-binding surface (blue) and non-ice-binding surface (red) are shown. Hydrogen bonding between helices stabilizes the fold.

**Figure 4:**
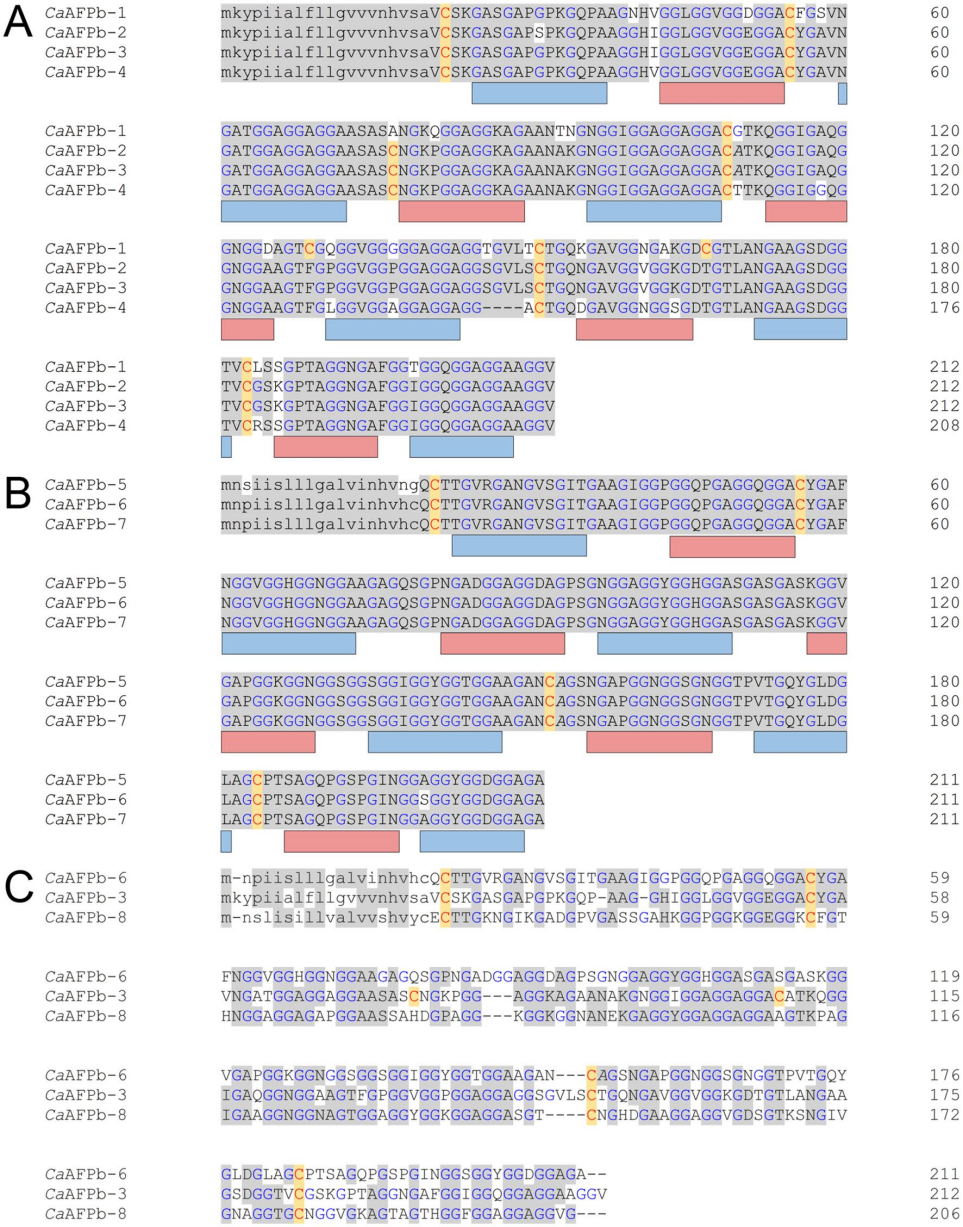
Sequence alignment of the 15-kDa *Ca*AFP isoforms. The amino acid sequences of *Ca*AFP isoforms from the generated transcriptome were aligned for the (**A**) first and (**B**) second groups. (**C**) The third *Ca*AFP isoform (*Ca*AFPb-8) is aligned to one sequence from the first group (*Ca*AFPb-3) and the second group (*Ca*AFPb-6). The colouring is the same as in Fig. 3. *Ca*AFPb-1, OQ445590; *Ca*AFPb-2, OQ445584; *Ca*AFPb-3, OQ445585; *Ca*AFPb-4, OQ445589; *Ca*AFPb-5, OQ445588; *Ca*AFPb-6, OQ445592; *Ca*AFPb-7, OQ445593; *Ca*AFPb-8, OQ445591.

There are over 3400 *C. antarcticus* ESTs, from two studies^42,43^, in the NCBI database. BLAST searches identified two with full-length coding sequences (GR869204.1 and FF279148.1), and two with incomplete coding sequences (GR870234.1 and FF278983.1). The full-length transcripts encoded a 108-amino-acid protein (8.5 kDa) after the removal of the 19-amino-acid signal peptide (Fig. 3A). GR870234.1 was missing the N-terminal methionine start codon and FF278983.1 had a truncated C terminus at residue 86. Peaks with similar masses were seen in the MALDI profile (Fig. 2B). Additional isoforms were identified within the transcriptome generated in this study; three that encode 8.5-kDa isoforms (OQ445583, OQ445586, and OQ445587) and eight that encode 15-kDa isoforms (OQ445584, OQ445585, OQ445588, OQ445589, OQ445590, OQ445591, OQ445592, OQ445593). The 8.5-kDa isoforms had between 91 and 93% sequence identity at the nucleotide and protein levels (Fig. 3A). Using a schematic to visualize each helix the 8.5-kDa isoforms can be modelled to have 6 helices (Fig. 3B). The strings of 3–4 GX_1_X_2_ repeat motifs can be separated into individual helices separated by variable loop regions that are 3–6 amino acid residues in length. The X_2_ residues in one helix and the following helix alternate between a hydrophobic and hydrophilic residue, and this produces the distinctive ice-binding site (Fig. 3 blue surfaces) and non-ice-binding site (Fig. 3 red surfaces). The 15-kDa isoforms clustered into three groups. The first group had three isoforms with a 20-amino-acid signal peptide and 192-amino-acid mature protein (Fig. 4A). *Ca*AFPb-4 had a deletion of four amino acids, making the mature protein 188 amino acids in length. The second group had three isoforms with a 19-amino-acid signal peptide and 192-amino-acid mature protein (Fig. 4B). The third type had a single isoform (*Ca*AFPb-8) with a 19-amino-acid signal peptide and a 187-amino-acid mature protein (Fig. 4C). Within the first and second groups, the isoforms showed between 88–99% and 98–99% sequence identity, respectively, while between these groups and the third isoform type there was only 50–52% sequence identity. Within each group, the amino acid sequences in the loop and non-IBS regions were less conserved between isoforms. When the three groups of the 15-kDa *Ca*AFPs are compared, the number of PPII helices was constant, with 11 predicted (Fig. 4), and the length of each helix was roughly equivalent, with three to four GXX or GGX repeats each. Additionally, the number of disulfide bonds predicted varied, from 2 to 4. Predicted average masses of *Ca*AFPb-8 (15.2 kDa) and *Ca*AFPb-6 (15.5 kDa) closely match peaks 15,158 and 15,463 Da seen by MALDI (Fig. 2B).

**Figure 5:**
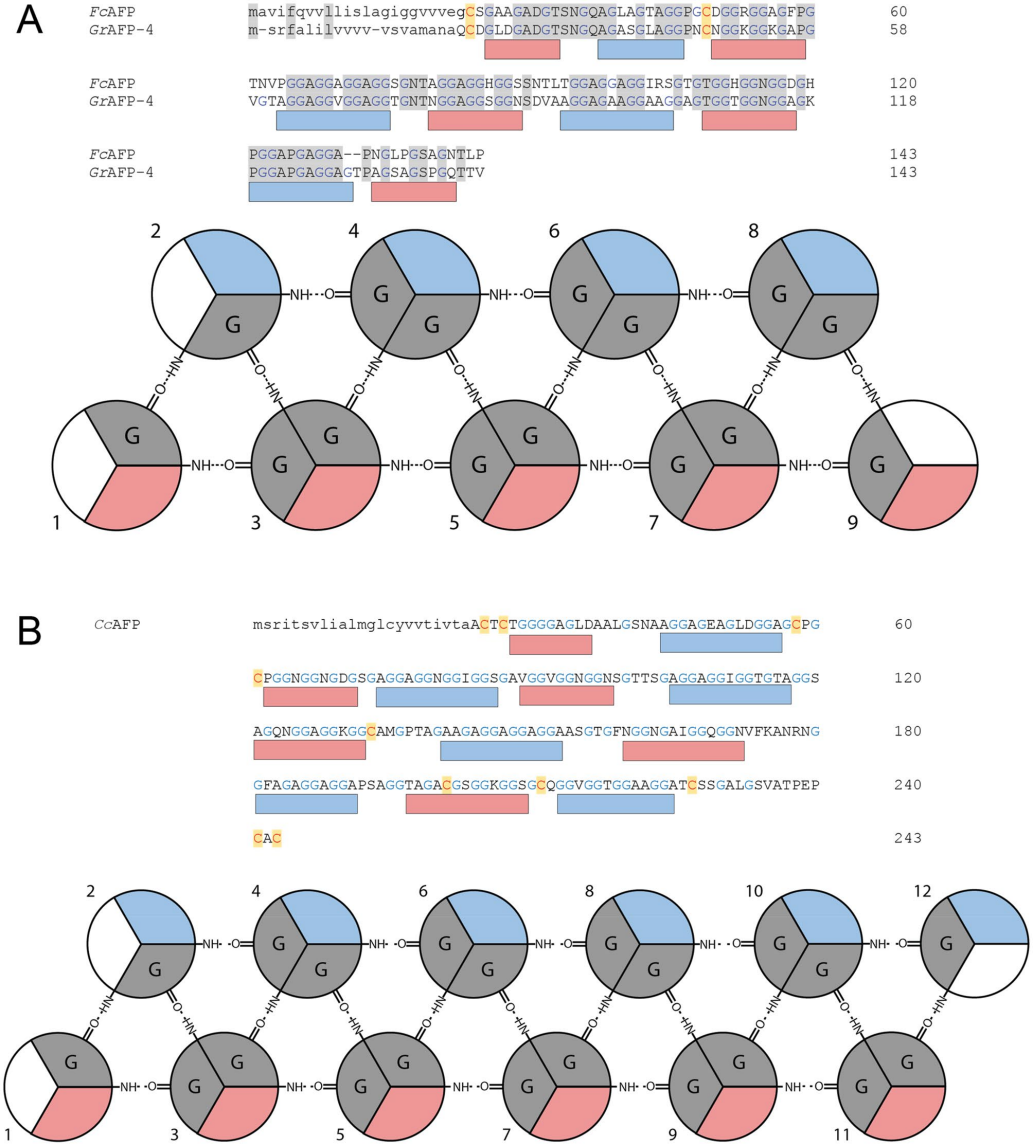
Polyproline type II helical bundle schematics. The protein sequences of *Fc*AFP and *Cc*AFP arranged into individual polyproline helices. (**A**) *Fc*AFP can be arranged into 9 helices and (**B**) *Cc*AFP can be arranged into 12 helices Colouring is the same as in Fig. 3.

The annotated assembly of the genome of *F. candida*^44^ was found to contain a single gene encoding a glycine-rich sequence (OXA44825.1), herein called *Fc*AFP, resembling other PPII helical AFPs. *Fc*AFP shares 57% sequence identity with *Gr*AFP-4 (Fig. 5A). *Fc*AFP was predicted to have a structure very similar to that of GrAFP-4 solved by crystallography^39^, with nine PPII helices forming an ice-binding face made up of the four even-numbered helices.

The tryptic fragments analyzed by tandem mass spectrometry (Table 1) support the contention that *F. candida*, unlike the other species examined, has but one AFP isoform. The dominant spectra matched the three predicted tryptic fragments, and they were sequenced multiple times, over most of their length. As the genome^44^ and this sample (Supplementary Table 1) were derived from the same parthenogenetic laboratory strain, this exact match was not unexpected. Similar fragments of different masses arose either from in-source fragmentation of the tryptic fragment^45^, or via post-translational modification. Fragments were sometimes 16 Da heavier than expected, with the additional mass coinciding with proline residues that were followed by glycine residues (Table 1, in bold).

The gene sequence and tryptic fragment sequences were consistent with the masses observed by MALDI-MS. The dominant peak was at 9646 m/z, with the double-charged species at 4832 m/z, but close examination of the spectrum (Fig. 2E, inset) reveals four peaks differing by ~ 16 Da. The lightest, at 9632 m/z, closely matches the average mass of 9630 Da predicted for the mature protein without modifications, whereas the others at 9464, 9663 and 9679 likely contain 1, 2 or 3 modified proline, respectively. A mass increase of 16 Da is consistent with hydroxylation of proline. Collagen contains X_1_-X_2_-G repeats and the structure consists of three PPII helices in parallel that form the collagen triple helix^46^. Proline is modified to hydroxyproline within X-P-G motifs throughout^47^. Therefore, it is likely that the same process is responsible for modifying, on average, one of the six X-P-G motifs in the AFP, given that the sequence repeats and secondary structure of the two proteins are similar.

The extracted protein from *F. candida* had only 0.2 °C of TH activity at the concentration tested, lower than most other species (Fig. 1). When recombinantly expressed in *E. coli, Fc*AFP reached 0.52 ± 0.01 °C of TH at a concentration of only 2.3 μM. This suggests that even after cold acclimation, the levels of AFP produced in the animal from this single gene are well below what can be attained in vitro. Despite producing an AFP, when *F. candida* were exposed to − 3 °C for 15 days none of specimens survived^32^. However, when starved their supercooling point is below − 15 °C^48^. Arthropods with high TH activity generally have more than one AFP gene, producing different AFP isoforms, as exemplified by the spruce budworm moth with its 16 gene copies^49^, and the other springtails herein.

*Entomobrya nivalis* was previously known to have up to 3.5 °C TH activity within their hemolymph during the winter months^50^. Therefore, the limited number of animals that were collected in the field were acclimated, before their RNA was extracted. A transcriptome was generated from which five AFP isoforms were identified (Supplementary Fig. 1B). Each sequence had a signal peptide between 22 and 24 amino acids in length. The mature proteins had predicted masses of 8.9 kDa, 9.6 kDa, and 11.2 kDa. The 8.9-kDa isoform (*En*AFP-2) was 86% identical to the 11.2-kDa isoform (*En*AFP-3) with a 29-amino-acid deletion that likely removes two helices. The other sequences had between 64 and 81% identity.

Using the sequences of other collembolan AFPs as a BLAST query, a sequence resembling a PPII helical AFP was found in the genome of *Ceratophysella communis* (VNWX01004235.1) The gene was predicted to contain a single intron^51^, and the resulting 732-bp open reading frame encoded an AFP predicted to have a 23-amino-acid signal peptide^52^. The mature protein was 220 amino acids in length and can be modelled to have 12 PPII helices (Fig. 5B). Although *C. communis* was not collected, *C. denticulata* was, and the homogenate had 0.7 °C TH (Fig. 1). Additionally, the largest peak on the MALDI-MS had a mass of 17.5 kDa, similar to the 17.2 kDa predicted for the mature *C. communis* AFP sequence.

### Polyproline AFPs are spread across orders of Collembola

**Figure 6:**
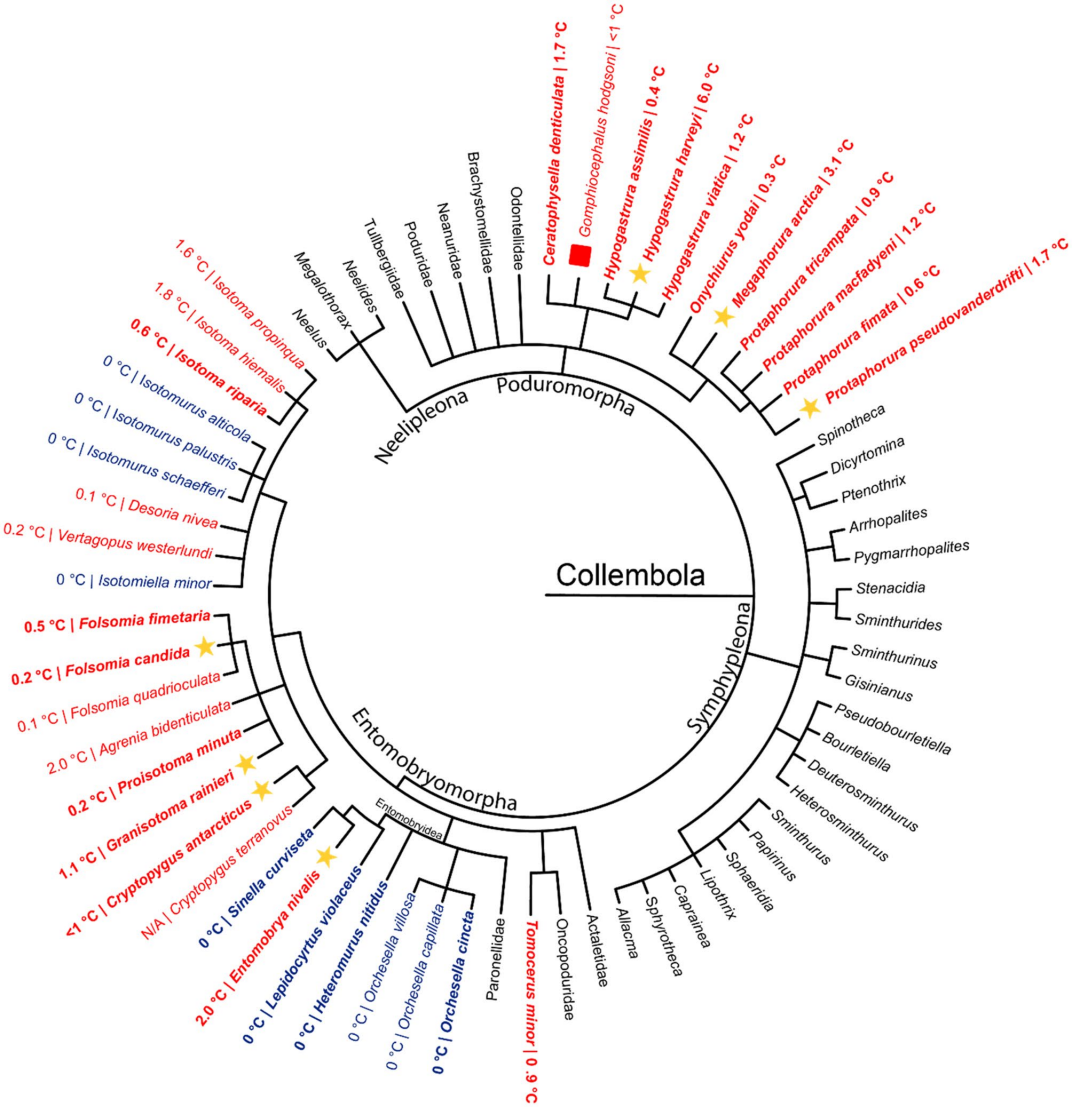
Taxonomic tree of antifreeze-protein-producing Collembola. A taxonomic tree for species from the four orders of Collembola (Entomobryomorpha, Poduromorpha, Symphypleona, and Neelipleona) was generated based on NCBI taxonomy. Species with and lacking TH activity are coloured in red and blue, respectively, and groups not tested are in black. Species assayed in this paper are bolded and all other species not bolded were tested by Zettel^28^, except *Gomphiocephalus hodgsoni*^30^ and *Cryptopygus terranovus* (syn. *Gressittacantha terranova*)^53^. Species with a star produced glycine-rich AFPs. *Gomphiocephalus hodgsoni* with a red square is a proposed cystine- and histidine-rich AFP^30^.

Ten of the collembolan species sampled were from Poduromorpha and 12 were from Entomobryomorpha (Fig. 6). All ten poduromorphs and eight entomobryomorphs had AFP activity. The four species lacking AFP activity were from the family Entomobryidae, but *E. nivalis* was inside this family as well and it did have AFP activity. Fourteen other species have been tested for TH activity by others^28,30,53^, for a total of 11 of 11 poduromorphs and 15 of 25 entomobryomorphs testing positive for AFP activity. Of the species that did not produce AFPs, two were again found in Entomobryidae, as well as four from Isotomidae.

**Figure 7:**
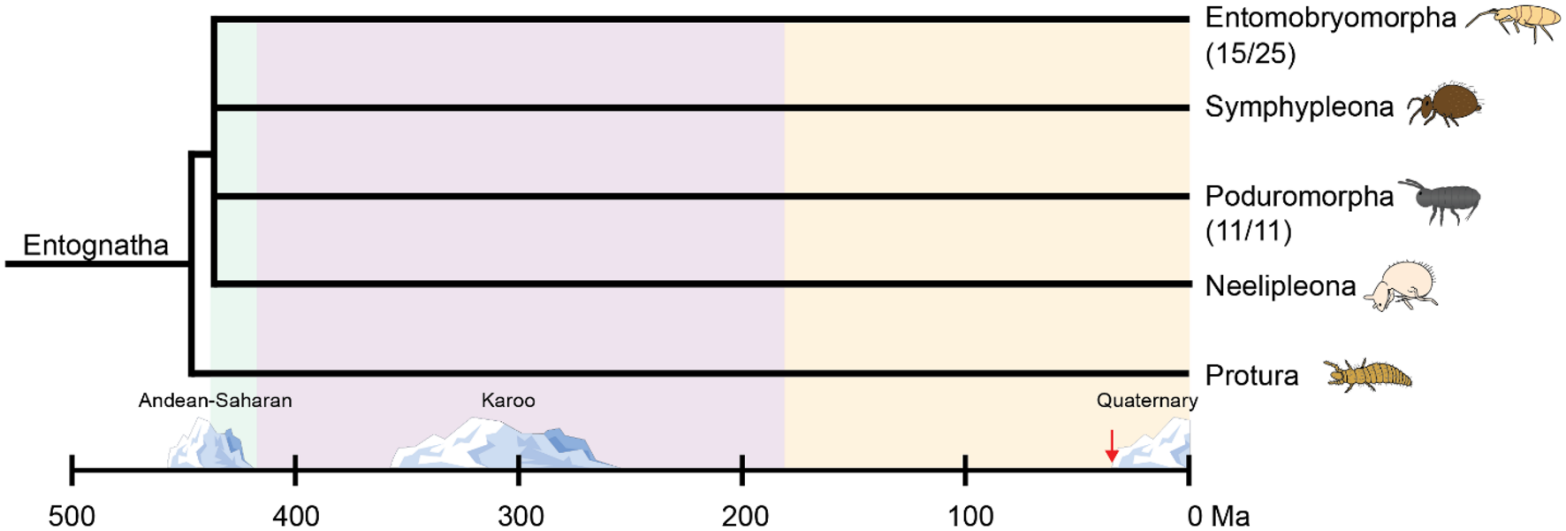
The relationship between collembolan phylogeny and ice ages. A phylogenetic tree showing the timeline of divergence in Collembola. The shaded green, purple, and yellow show estimated times for divergence of orders, families, and genera/ species, respectively. Number of AFP-producing species are shown below the order name. The red arrow indicates the emergence of fish AFPs during the Cenozoic era. Icebergs display the timespan of each respective ice age.

The glycine-rich PPII helical bundle was predicted to be the AFP fold of eight species of Collembola, spanning five families and two orders. The presence of PPII helical AFPs in both Entomobryomorpha and Poduromorpha suggests that this protein family originated prior to their divergence (Fig. 7). The exact sequence of collembolan taxonomic diversification is still being debated. The four orders can be arranged into assorted sister clades depending on the datasets used. When using *18S* and *28S* sequences Neelipleona was basal to (Symphypleona + (Entomobryomorpha + Poduromorpha)^54^. Yet, when using *16S, 28S, and cox1* sequences the positions of Neelipleona and Symphypleona were reversed and Symphypleona was basal^55^. Regardless of the exact phylogenetic relationship, mitochondrial dating suggests that the four orders diverged between 437 and 421 million years ago^56^. This period coincides with the Andean-Saharan glaciation, an ice age that lasted from 460 to 420 million years ago^57^. Additionally, the lineages corresponding to extant families diverged between 414 and 184 million years ago^56^, in which the Karoo glaciation occurred, between 360 and 255 million years ago^58^. Diversification and the need for freeze resistance in the same timeframe would allow for radiation of the species expressing PPII helical AFP.

The timing of diversification into genera could have led to species lacking AFPs. Within the superfamily Entomobryoidea, *Sinella curviseta*, *Heteromurus nitidus*, *Lepidocyrtus violaceus* and three species from the genus *Orchesella* did not display antifreeze activity AFPs, while *E. nivalis* did (Fig. 6). Analyses of a sample of Collembola from China estimated that five polyphyletic clades of the genus *Entomobrya* diversified between 66 and 34 million years ago during the Paleocene–Eocene thermal maximum^59^. During this period, around 55 million years ago, global temperatures were an average of 5–8 °C higher than today^60^. *Sinella* is estimated to have diverged from one *Entomobrya* clade around 69 million years ago, while *Heteromurus* and *Orchesella* diverged from the family *Entomobryidae* around 100 million years ago^59^. The ancestor of the AFP-lacking species in the superfamily Entomobryoidea might have lost their AFP during this period, leading to radiation of species without an AFP gene, while the *E. nivalis* lineage retained theirs.

Unlike teleost fish, where AFPs did not originate until the Cenozoic era (~ 30 million years ago) following the Paleocene–Eocene thermal maximum^20^, there is no sign of a sudden diversification of collembolan AFPs during this event or the previous Karoo glaciation. In theory, certain lineages may have lost their PPII AFP type gene(s) during an interglacial period, only to evolve a replacement to cope with a new ice age. For this reason, it is worth noting the different amino acid composition of an AFP previously identified in the Antarctic collembolan species, *Gomphiocephalus hodgsoni*^30^. This AFP contained high percentages of histidine and cystine that set it apart from the PPII-type AFPs (Supplementary Table 2). However, the sequence of this AFP has not yet been identified. Interestingly, this species is a member of the Hypogastruridae family in which two species (*H. harveyi* and *C. communis*) produce glycine-rich PPII helical AFPs.

The origins and relatedness of the PPII AFPs are difficult to determine as the repetitive nature of the protein makes comparisons between distantly related species extremely difficult. Homology cannot be easily inferred from repetitive sequences, especially when they are under selection for antifreeze activity. For example, the alanine-rich type I AFPs of fish, some of which have threonine residues at 11-amino-acid intervals, initially appeared homologous, but they are now known to have evolved via convergence within the last 30 million years^15^. Fortunately, the origin of the flounder type I AFPs was traced via their UTRs^20^. It is possible that convergence also played a role in the evolution of collembolan AFPs. However, this seems unlikely, as all but one species examined to date produce glycine-rich AFPs and the known sequences form^34,39^, or can be modelled (Figs. 3, 5)^31,38^ as, PPII helical bundles with an ice-binding face. In contrast, when AFPs arose in teleost fishes and insects, a variety of different proteins folds were used as AFPs^61–65^. Additionally, the length of the PPII helices do not vary between isoforms or species. When an extra GGX repeat was added to each helix of *Gr*AFP the TH activity decreased suggesting that this could be a selective pressure to limit the length of the helices^37^.

It is unlikely that an analysis of the UTRs of collembolan AFPs will provide clues as to their origins. Many of the species studied herein diverged much earlier than teleost fish (Fig. 7). This has provided ample time for their non-coding regions to diverge to such an extent as to be unrecognizable as homologous. This is evident even when the 5ʹ- and 3ʹ-UTRs are compared between transcripts from a single species. For example, the 5ʹ- and 3ʹ-UTRs of *En*AFPs have between 65–93% and 60–84% sequence identity, respectively. The lack of non-coding sequence identity has been previously reported between *Hh*AFP and *Ma*AFP^31^, and between isoforms of *Hh*AFP^38^ and *Gr*AFP^39^. This suggests that some of these PPII AFPs, even those from the same species, have been diverging for far longer than 30 million years.

One limitation of this study was our inability to sample species from either Neelipleona or Symphypleona. There is currently an underrepresentation of genomic and transcriptomic data for these orders relative to Entomobryomorpha and Poduromorpha, but nine genome sequences are publicly available at NCBI. Although PPII AFPs were not found in the eight Symphypleona and one Neelipleona genome sequences, it should be noted that the repetitive nature of the PPII AFPs, along with the abundance of glycine-rich genes (such as collagen), introns, and repetitive sequences replete with potential glycine codons, make identification of AFP genes difficult. Therefore, identification of these AFPs is heavily reliant on tissue extraction. Unfortunately, to the best of our knowledge, laboratory culturing of Symphypleona and Neelipleona is difficult, complicating detailed studies on AFPs in these two orders.

## Supplementary Information

Supplementary Information.

## Data Availability

The datasets generated during the current study are available in the GenBank repository (https://www.ncbi.nlm.nih.gov/genbank/) under the accession numbers OQ511494-98 and OQ445583-93.
