# Supplementary material for: Polyproline type II helical antifreeze proteins are widespread in Collembola and likely originated over 400 million years ago in the Ordovician Period
Source: Sci Rep. 2023 Jun 1;13:8880. doi: 10.1038/s41598-023-35983-y (PMC10235112; doi:10.1038/s41598-023-35983-y)
Supplement: Supplementary file 1 — Supplementary Information. [file 41598_2023_35983_MOESM1_ESM.pdf]

Supplementary Table 1. Collection details for collembolan specimens

Superfamily	Family	Species	Region of collection	Time in culture (approximately)	Latitude	Longitude
Poduromorpha	Hypogastruridae	*Hypogastrura assimilis*	Rønde, Denmark	18 y	56°29 N	10°48 E
		Hypogastrura viatica	Ny Aalesund, Svalbard	4 y	78°93 N	11°87 E
		Ceratophysella denticulata	Jægerspris, Denmark	4 y	55°86 N	11°97 E
	Onychiuridae	*Protaphorura fimata*	Göttingen, Germany	20 y	51°49 N	9°84 E
		Protaphorura macfadyeni	Nuuk, Greenland	4 y	64°20 N	51°42 W
		Protaphorura pseudovanderdrifti	Hveragerði, Iceland	3 y	64°00 N	21°10 W
		Protaphorura tricampata	Jægerspris, Denmark	6 y	55°86 N	11°97 E
		Megaphorura arctica	Sauðárkrókur, Iceland	field collected	65°52 N	19°44 W
		Onychiurus yodai	Shanghai, China	5 y	31°36 N	120°28 E
Entomobryomorpha	Isotomidae	*Folsomia candida*	Berlin, Germany	25 y	52°38 N	13°34 E
		Folsomia fimetaria	Askov, Denmark	20 y	55°27 N	9°05 E
		Proisotoma minuta	Jægerspris, Denmark	4 y	55°86 N	11°97 E
		Isotoma riparia	Amsterdam, The Netherlands	1 y		
	Entomobryidae	*Sinella curviseta*	Vancouver, Canada	15 y	50°08 N	123°01 W
		Orchesella cincta	Alès, France	3 y	44°08 N	3°59 E
		Heteromurus nitidus	Rome, Italy	15 y	41°59 N	12°35 E
		Lepidocyrtus violaceus	Silkeborg, Denmark	1 y		
		Entomobrya nivalis	Rønde, Denmark	field collected	56°29 N	10°48 E
		Cryptopygus antarcticus	Joubin Islands, Antarctica	field collected	64°47 S	64°26 W
	Tomoceridae	*Tomocerus minor*	Silkeborg, Denmark	1 y	56°15 N	9°56 E

Supplementary Table 2. Amino acid composition of ice-affinity purified collembolan AFP extracts.

SPECIES	Gly	Ala	Other
Hypogastrura harveyi[29] [a]	45.6	13.9	40.5
Granisotoma rainieri[39]	22.3	12.6	65.1
Cryptopygus antarcticus	31.9	11.7	56.4
Folsomia candida	26.9	11.1	62.0
Protaphorura pseudovanderdrifti	23.1	14.9	62.0
Megaphorura arctica[31]	36	13	51
Gomphiocephalus hodgsoni[30]	11.5	6.9	81.6

[a]This *Hh*AFP was further purified by high-performance liquid chromatography. The most abundant amino acids in *Gh*AFP were and Cys (13.8%) and His (11.5%).

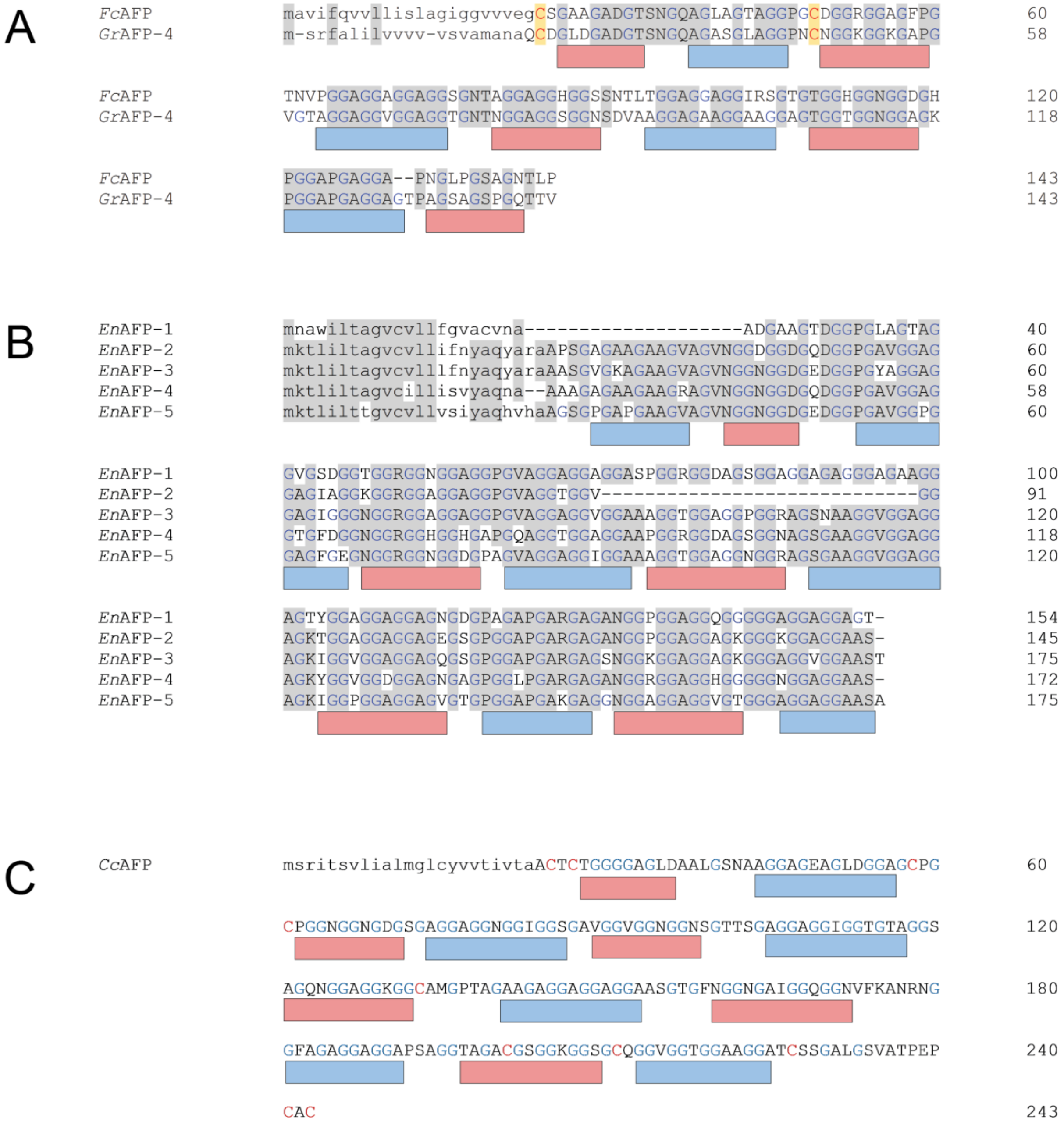

Supplementary Figure 1. *Fc*AFP ,*Gr*AFP, *En*AFP, and *Cc*AFP sequences. A) The amino acid sequences of *Fc*AFP (OXA44825.1) and *Gr*AFP-4 (QQY00623.1) were aligned B) The five isoforms of EnAFP (OQ511494 – 98) were aligned. C) The sequence of *Cc*AFP (from VNWX01004235.1). Colouring is the same as in Fig. 3
